# Poly(ADP-ribosyl)ation of acetyltransferase NAT10 by PARP1 is required for its nucleoplasmic translocation and function in response to DNA damage

**DOI:** 10.1186/s12964-022-00932-1

**Published:** 2022-08-19

**Authors:** Hong-Yi Liu, Ying-Ying Liu, Yin-Ling Zhang, Yan Ning, Fang-Lin Zhang, Da-Qiang Li

**Affiliations:** 1 Fudan University Shanghai Cancer Center and Institutes of Biomedical Sciences, Fudan University, Shanghai, 200032 China; 2 Cancer Institute, Shanghai Medical College, Fudan University, Shanghai, 200032 China; 3 Department of Oncology, Shanghai Medical College, Fudan University, Shanghai, 200032 China; 4 Department of Breast Surgery, Shanghai Medical College, Fudan University, Shanghai, 200032 China; 5 Department of Pathology, Obstetrics and Gynecology Hospital, Fudan University, Shanghai, 200032 China; 6 Shanghai Key Laboratory of Breast Cancer, Shanghai Medical College, Fudan University, Shanghai, 200032 China; 7 Shanghai Key Laboratory of Radiation Oncology, Shanghai Medical College, Fudan University, Shanghai, 200032 China

**Keywords:** DNA damage response, Nucleoplasmic translocation, Nucleolar localization signal, Posttranslational modification, PARylation

## Abstract

**Background:**

N-acetyltransferase 10 (NAT10), an abundant nucleolar protein with both lysine and RNA cytidine acetyltransferase activities, has been implicated in Hutchinson-Gilford progeria syndrome and human cancer. We and others recently demonstrated that NAT10 is translocated from the nucleolus to the nucleoplasm after DNA damage, but the underlying mechanism remains unexplored.

**Methods:**

The NAT10 and PARP1 knockout (KO) cell lines were generated using CRISPR-Cas9 technology. Knockdown of PARP1 was performed using specific small interfering RNAs targeting PARP1. Cells were irradiated with γ-rays using a ^137^Cs Gammacell-40 irradiator and subjected to clonogenic survival assays. Co-localization and interaction between NAT10 and MORC2 were examined by immunofluorescent staining and immunoprecipitation assays, respectively. PARylation of NAT10 and translocation of NAT10 were determined by in vitro PARylation assays and immunofluorescent staining, respectively.

**Results:**

Here, we provide the first evidence that NAT10 underwent covalent PARylation modification following DNA damage, and poly (ADP-ribose) polymerase 1 (PARP1) catalyzed PARylation of NAT10 on three conserved lysine (K) residues (K1016, K1017, and K1020) within its C-terminal nucleolar localization signal motif (residues 983–1025). Notably, mutation of those three PARylation residues on NAT10, pharmacological inhibition of PARP1 activity, or depletion of PARP1 impaired NAT10 nucleoplasmic translocation after DNA damage. Knockdown or inhibition of PARP1 or expression of a PARylation-deficient mutant NAT10 (K3A) attenuated the co-localization and interaction of NAT10 with MORC family CW-type zinc finger 2 (MORC2), a newly identified chromatin-remodeling enzyme involved in DNA damage response, resulting in a decrease in DNA damage-induced MORC2 acetylation at lysine 767. Consequently, expression of a PARylation-defective mutant NAT10 resulted in enhanced cellular sensitivity to DNA damage agents.

**Conclusion:**

Collectively, these findings indicate that PARP1-mediated PARylation of NAT10 is key for controlling its nucleoplasmic translocation and function in response to DNA damage. Moreover, our findings provide novel mechanistic insights into the sophisticated paradigm of the posttranslational modification-driven cellular response to DNA damage.

**Video Abstract**

**Supplementary Information:**

The online version contains supplementary material available at 10.1186/s12964-022-00932-1.

## Background

Lysine acetyltransferases (KATs) are a highly diverse group of enzymes responsible for transferring an acetyl group from acyl coenzyme A to a lysine residue on histones and non-histone proteins, and play a central role in the regulation of gene transcription, DNA damage response, metabolism, and cell signaling [1–3]. To date, 22 different KATs have been identified in the human genome, which be grouped into three main families based on their catalytic domains, including general control non-repressible 5 (GCN5)-related N-acetyltransferase (GNAT), p300/CREB-binding protein (p300/CBP), and MOZ, YBF2, SAS2, and TIP60 (MYST) families [2, 3]. N-acetyltransferase 10 (NAT10) is a unique member of the GNAT family of KATs, which possesses both RNA cytidine acetyltransferase and lysine acetyltransferase activities [4–7]. As an RNA cytidine acetyltransferase, NAT10 catalyzes the formation of N4-acetylcytidine (ac4C) modification of mRNA, rRNA, and tRNA to regulate ribosome biogenesis [6–10]. In addition, NAT10 participates in multiple cellular processes through its lysine acetyltransferase activity toward non-ribosomal targets, such as histones [11], α-tubulin [12], centrosome protein CCDC84 [13], tumor suppressor p53 [14], and autophagy regulator Che-1 [15]. Recently, we discovered MORC family CW-type zinc finger 2 (MORC2), a newly identified chromatin-associated enzyme involved in the DNA damage response (DDR), as a novel acetylated substrate of NAT10 [16]. NAT10-mediated MORC2 acetylation renders breast cancer cells resistant to DNA-damaging chemotherapy and radiotherapy by activating cell-cycle checkpoint control [16]. In addition to acetylating a variety of substrates, NAT10 undergoes autoacetylation, which is critical for its function in rRNA transcription activation [15, 17].

NAT10 contains at least one nuclear localization signal (NLS) and one possible nucleolar localization signal (NuLS) motif at its N-terminus, and one NLS and NuLS at its C-terminus [18]. Accumulating evidence shows that NAT10 is predominantly localized in the nucleolus [12, 19], and its mislocalization has been linked to human cancer progression [20–22]. Interestingly, we and others recently demonstrated that NAT10 translocates to the nucleoplasm from the nucleolus upon DNA damage [14, 16], but the underlying mechanism remains unexplored.

In vertebrate cells, the DNA damage response (DDR) is primarily controlled by poly(ADP)ribose polymerase 1 (PARP1) and three protein kinases of the phosphatidylinositol 3-kinase related kinase (PIKK) family, including ataxia telangiectasia mutated (ATM), ataxia telangiectasia and Rad3-related (ATR), and DNA-dependent protein kinase catalytic subunit (DNA-PKcs) [23, 24]. PARP1 is a highly conserved DNA damage-dependent enzyme that accounts for approximately 90% of total cellular PARP activity and orchestrates early DDR events by catalyzing the synthesis of poly(ADP-ribose) (PAR) at sites of DNA damage [25]. PAR can be covalently attached to acceptor proteins, a widespread posttranslational modification (PTM) known as poly(ADP-ribosyl)ation (PARylation), or mediate the recruitment of DNA repair factors bearing PAR-binding modules to sites of DNA lesions via noncovalent interactions [24, 25]. In contrast, PIKK protein kinases trigger the DDR signaling cascade by phosphorylating their downstream substrates [26]. Despite these advances, the biological functions and related mechanisms of these enzymes in sensing and responding to genotoxic stress remain largely unknown.

In this study, we report for the first time that PARP1-mediated PARylation is a novel post-translational modification of NAT10, which is essential for its translocation from the nucleolus to the nucleoplasm and for acetylating its substrate, MORC2, in response to DNA damage. These findings highlight a coordinated mechanism for multiple DNA damage-related enzymes in the regulation of cellular responses to DNA damage.

## Materials and methods

### Cell culture and treatment

Human breast cancer cell lines MCF-7 (#SCSP-531) and BT549 (#TCHu 93) and human embryonic kidney cell line HEK293T (#SCSP-502) were provided by the Cell Bank of the Chinese Academy of Sciences (Shanghai, China) and Shanghai Key Laboratory of Breast Cancer (Fudan University), and were authenticated by detection of mycoplasma and cell vitality, and short tandem repeat (STR) profiling. Cells were cultured in DMEM (BasalMedia, #L110) supplemented with 10% fetal bovine serum (ExCell Bio, #FSP500) and 1 × penicillin–streptomycin solution (BasalMedia, #S110B). Exponentially growing cells were irradiated with γ-rays at a dose rate of 0.75 Gy/min using a ^137^Cs Gammacell-40 irradiator (Institute of Radiation Medicine, Fudan University) at room temperature as described previously [16]. Detailed information for chemical inhibitors is provided in Additional file 1: Table S1.

### Expression vectors, plasmid transfection, and lentiviral infection

Myc-DDK-tagged MORC2 (Origene, #RC200518), Flag-His-NAT10 (Vigene, #CH874058), and GFP-tagged NAT10 (Origene, #RG207082) cDNAs have been described previously [16]. Molecular cloning was performed using either the ClonExpress Ultra One Step Cloning Kit (Vazyme, #C115-02) or CloneEZ PCR Cloning Kit (Genscript, #L00339). Amino-acid substitutions and deletion mutants were generated using PCR-directed mutagenesis. All construct sequences were verified by DNA sequencing. Detailed information concerning the expression constructs and the primers used for molecular cloning is provided in Additional file 1: Tables S2 and S3.

Transient plasmid transfection was performed using the Neofect DNA transfection reagent (TengyiBio, #TF201201) according to the manufacturer's protocol. Lentiviral infection and generation of stable cell lines were carried out as described previously [27, 28]. The NAT10 and PARP1 knockout (KO) cell lines were generated as described previously [29] and validated by immunoblotting analysis and Sanger sequencing. Individual gRNA sequences have been described previously [16].

### Small interfering RNAs (siRNAs) and transfection

SiRNAs targeting PARP1 (siPARP1) and corresponding negative control siRNA (siNC) were obtained from GenePharma (Shanghai, China). The siRNA targeting sequences are listed in Additional file 1: Table S4. Lipofectamine 2000 transfection reagents (Invitrogen, #2041726) were used to transfect siRNA duplexes into cells according to the manufacturer’s instructions. Knockdown efficiency of siRNAs was verified by immunoblotting 48 h after transfection.

### Antibodies, immunoblotting, and immunoprecipitation assays

All antibodies used in this study are listed in Additional file 1: Table S5. Immunoblotting and immunoprecipitation (IP) assays [16, 30] and Dot blotting assays [31] were performed as described previously. The optical density of the immunoblotting bands was quantified using ImageJ software and was normalized to the internal control vinculin.

### Purification of recombinant proteins

The GST-tagged NAT10 fragments in the pGEX-6P-1 vector were transformed into the *E. coli* strain BL21 (DE3), incubated with 0.2 mM IPTG (Invitrogen, #15529019) at 16 °C overnight, and then purified using Glutathione Sepharose 4B beads (GE Healthcare, #17075601) according to the manufacturer's instructions. The purified proteins were immediately used for the experiments or frozen at − 80 °C.

### In vitro PARylation assays

The purified GST-NAT10 fragment (1 μg) was incubated with 100 ng of recombinant full-length PARP1 (Origene, #TP710053) in a reaction buffer containing 100 mM Tris–HCl (pH 8.0), 10 mM MgCl_2_, 1 mM DTT, 4 ng/ml sonicated salmon sperm DNA (Invitrogen, #AM9680), and 300 μM nicotinamide adenine dinucleotide (NAD^+^) at 37 °C for 30 min. The reaction was terminated by the addition of 2 × SDS loading buffer, and PARylation of NAT10 was detected by immunoblotting with an anti-PAR monoclonal antibody (Trevigen, #4335-MC-100).

### Immunofluorescent staining

Immunofluorescence staining was performed as described previously [27, 28]. Briefly, cells were fixed with 4% methanol-free formaldehyde (Yeasen, #36314ES76) for 20 min at room temperature and permeabilized with 0.5% Triton X-100 for 20 min at 4 °C. After three rinses with PBS, cells were blocked with 5% goat serum for 1 h at room temperature and incubated with anti-HA (1:500) and anti-Flag (1:500) antibodies in 5% goat serum overnight at 4 °C. Cells were rinsed three times with PBS and incubated with secondary antibodies conjugated with Alexa 488 or Alexa-568 (1:500) at room temperature for 1 h. After washing three times with PBS, the cells were sealed with DAPI-containing fluoroshield mounting medium (#ab104139; Abcam). Images were visualized using a Leica SP5 confocal microscope and analyzed.

### Clonogenic survival assays

A total of 5 × 10^3^ cells were seeded in 12-well plates in triplicate overnight. Then, cells were treated with increasing doses of the DNA-damaging agent methyl methanesulfonate (MMS) or were irradiated with γ-rays using a 137Cs Gammacell-40 irradiator. Cells were fixed after 10 days of treatment with methanol, stained with 0.2% crystal violet solution, and photographed. Colonies consisting of more than 50 cells were counted.

### Statistical analysis

All data are presented as the mean ± standard deviation of at least three independent experiments. The unpaired two-tailed Student’s *t-test* was used to compare data between two groups using SPSS20. Statistical significance was set at *p* < 0.05.

## Results

### NAT10 is a poly(ADP-ribosyl)ated protein in response to DNA damage

PARP1-mediated PARylation is well known to mediate the first wave of cellular response to DNA damage generated either exogenously or endogenously [25]. To determine whether NAT10 is modified by PARylation, MCF-7 and BT549 cells were treated with or without the DNA-damaging agents MMS and IR, and then subjected to IP assays with an anti-NAT10 antibody. Accumulating evidence shows that MMS is an alkylating agent that acts on DNA by preferentially methylating guanine and adenine bases to indue single strand breaks (SSBs) [32] as well as double-strand breaks (DSBs) [33, 34]. In contrast, IR primarily induce DSBs [35].

**Fig. 1:**
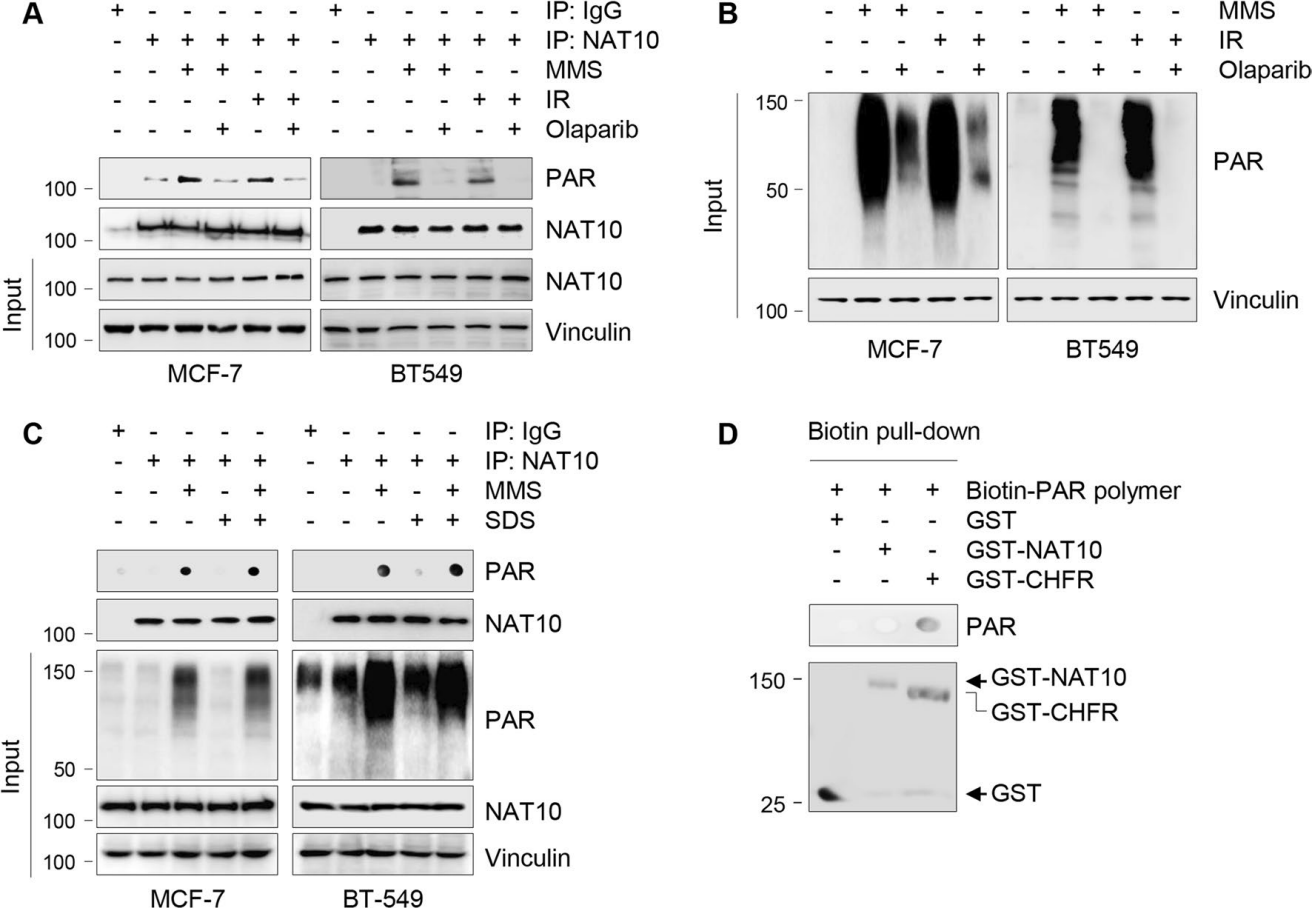
NAT10 is a novel substrate of PARylation in response to DNA damage. **A**, **B** MCF-7 and BT549 cells were pretreated with or without 10 μM Olaparib for 3 h, and then treated with or without 1 mM MMS for another 2 h or 6 Gy IR. Cells were harvested for IP and immunoblotting analyses with the indicated antibodies. **C** MCF-7 and BT549 cells were treated with or without 1 mM MMS for 2 h and subjected to IP analysis with an anti-NAT10 antibody. A total of 1% SDS was added to lysis buffer to remove all non-covalent binding. The immunoprecipitate was spotted onto a nitrocellulose membrane and the membrane was then examined using an anti-PAR antibody. Immunoblotting analysis was performed with anti-NAT10, PAR, and vinculin antibodies. **D** In vitro biotin pull-down assays were carried out by incubating purified GST-NAT10 or GST-CHFR with purified PAR (biotin-PAR polymer). GST-CHFR was used as a positive control

Immunoblotting analysis with an anti-PAR antibody revealed that treatment of cells with MMS and IR resulted in an increase in NAT10 PARylation, and the noted effects were significantly impaired by the pretreatment of cells with Olaparib, a potent PARP inhibitor (Fig. 1A). Similar to other PARylated substrates, such as transcription factor C/EBPβ [36], tumor suppressor protein p53 [37], and histone demethylase KDM4D [38], a single band was observed for PARylated NAT10. As a positive control, MMS and IR stimulated massive PAR formation, an indicator of PARP1 activation [39], which was significantly blocked in the presence of Olaparib (Fig. 1B).

In addition to covalent PARylation, PAR can bind non-covalently to target proteins with PAR-binding modules [25]. To exclude the possibility that NAT10 non-covalently interacts with PAR, we treated MCF-7 and BT549 cells with or without MMS and then immunoprecipitated endogenous NAT10 with an anti-NAT10 antibody in the absence or presence of sodium dodecyl sulfate (SDS), which could abolish the non-covalent interactions [40]. Dot blotting assays with an anti-PAR antibody showed that PARylation of NAT10 induced by MMS was not affected in the presence of SDS (Fig. 1C). To further validate these results, we performed in vitro pull-down assays by incubating recombinant GST-NAT10 or GST10-CHFR protein with purified PAR (biotin-PAR polymer) and then dot-blotted the pull-down complex on nitrocellulose membranes. Immunoblotting with an anti-PAR antibody revealed that GST-NAT10 did not bind to the biotin-PAR polymer in vitro (Fig. 1D). As a positive control, GST-CHFR directly bound to PAR, as reported previously [41] (Fig. 1D). Together, these findings suggest that NAT10 undergoes covalent PARylation following DNA damage.

### PARP1 PARylates NAT10 at K1016, K1017, and K1020 both in vitro and in vivo

**Fig. 2:**
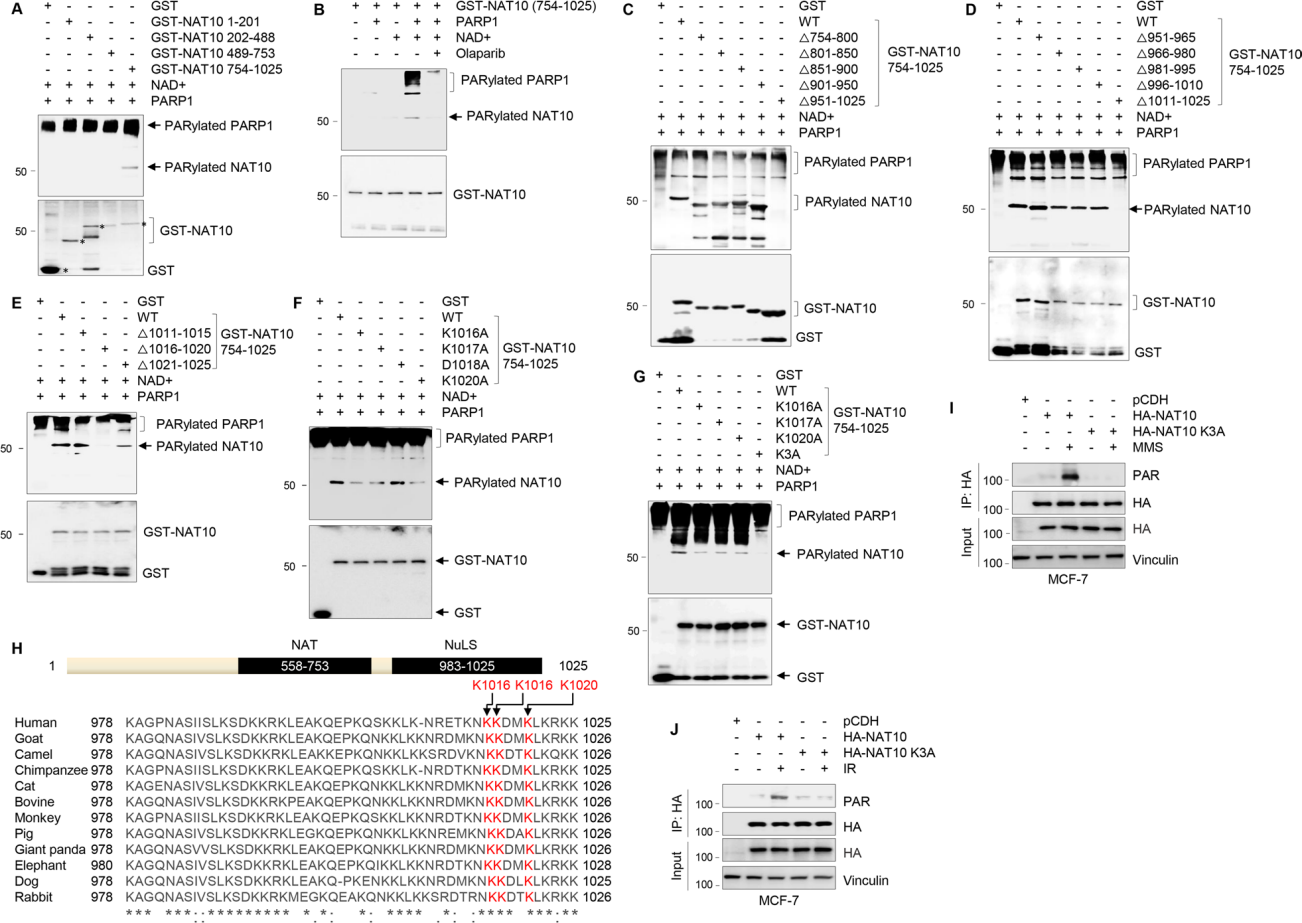
PARP1 PARylates NAT10 at K1016, K1017, and K1020 both in vitro and in vivo. **A** Four GST-NAT10 deletion mutants (∆1–201, ∆202–488, ∆489–753, and ∆754–1025) and GST control were bacterially purified, and subjected to in vitro PARation assay in the presence of PARP1 and NAD^+^. The reaction samples were resolved by SDS-PAGE, and analyzed by immunoblotting analyses with anti-PAR and anti-GST antibodies. **B** In vitro PARylation assays were performed using purified GST-NAT10 deletion fragments in the presence or absence of recombinant PARP1 enzyme, NAD^+^, and Olaparib. PARylated NAT10 was detected with an anti-PAR antibody. **C**–**E** GST-NAT10 deletion fragments were subjected to in vitro PARation assay as described in A. **F**, **G** Purified GST-NAT10 754–1025 proteins (WT, K1016A, K1017A, D1018A, and K1020A, K3A) were subjected to in vitro PARation assays in the presence of PARP1 and NAD^+^. PARylation of NAT10 was detected by immunoblotting with an anti-PAR antibody. In G, K3A represents the combined mutation in all three residues (K1016, K1017, and K1020). **H** Alignment of the NAT10 protein sequence among different organisms. Asterisk (*) indicates the full conservation of the residues of NAT10 among different species. **I**, **J** MCF-7 cells were transfected with HA-NAT10 or HA-NAT10 K3A expression vector. After 48 h of transfection, cells were treated with or without 1 mM MMS for 2 h (**I**) or 6 Gy IR (**J**). Thereafter, IP and immunoblotting analyses were conducted with the indicated antibodies

To map the PARylation sites of NAT10 by PARP1, we carried out in vitro PARylation assays using a series of GST-NAT10 deletion constructs. Of note, PARylation of full-length NAT10 was difficult to confirm by in vitro PARylation assays, as PARP1 undergoes auto-PARylation and NAT10 has an almost similar size to PARP1 (116 vs. 113 kDa). As shown in Fig. 2A, the C-terminal fragment of NAT10 (residues 754–1025, lane 5), but not other deletion fragments, was PARylated in the presence of nicotinamide adenine dinucleotide (NAD+) as a donor of ADP-ribose groups and recombinant PARP1 enzyme. Aligning with the notion that PARP1 is a major substrate of itself, PARP1 auto-PARylation was also observed in these assays. As a negative control, a reactive signal was not observed in the absence of either NAD^+^ (lane 2) or recombinant PARP1 (lane 3) (Fig. 2B). Moreover, the PARylation signal of NAT10 in the presence of NAD^+^ and recombinant PARP1 (lane 4) was reduced in the presence of PARP inhibitor Olaparib (lane 5) (Fig. 2B). These results indicate that NAT10 is primarily PARylated at its C-terminal region. Further mapping experiments using a series of small deletion constructs showed that a deletion mutant lacking amino acids 1016–1020 failed to be PARylated (Fig. 2C–E). Thus, the PARylation sites of NAT10 must reside within amino acids 1016–1020 (KKDMK).

As PARylation most commonly occurs on aspartate (D), glutamate (E), and lysine (K) residues of target proteins [24, 42, 43], we first substituted four residues (K1016, K1017, D1018, and K1020) with alanine (A) alone by site-directed mutagenesis, and found that a single mutation of K1016, K1017, and K1020 resulted in a reduction in NAT10 PARylation levels to a certain degree compared to its wild-type (WT) counterpart and D1018A mutation (Fig. 2F). Moreover, the combined mutations of three lysine residues (K1016A/K1017A/K1020A, termed K3A) resulted in no detectable PARylation of NAT10 (Fig. 2G), suggesting that NAT10 is primarily PARylated at K1016, K1017, and K1020. Interestingly, these PARylation residues in NAT10 are localized within its C-terminal nucleolar localization signal (NuLS) motif (residues 983–1025) [18]. Moreover, the K1016, K1017, and K1020 residues in NAT10 are highly conserved among different species (Fig. 2H).

To further validate the above results in vivo, MCF-7 cells were transfected with HA-NAT10 and HA-NAT10 K3A, and then treated with or without MMS and IR. IP and immunoblotting analysis using the indicated antibodies revealed that treatment with MMS and IR significantly enhanced PARylation of WT, but not the K3A mutant NAT10 (Fig. 2I, J). Together, these results suggest that NAT10 is primarily PARylated by PARP1 at the conserved K1016, K1017, and K1020 residues both in vitro and in vivo.

### PARylation of NAT10 by PARP1 controls its nucleoplasmic translocation and co-localization with MORC2 in response to DNA damage

**Fig. 3:**
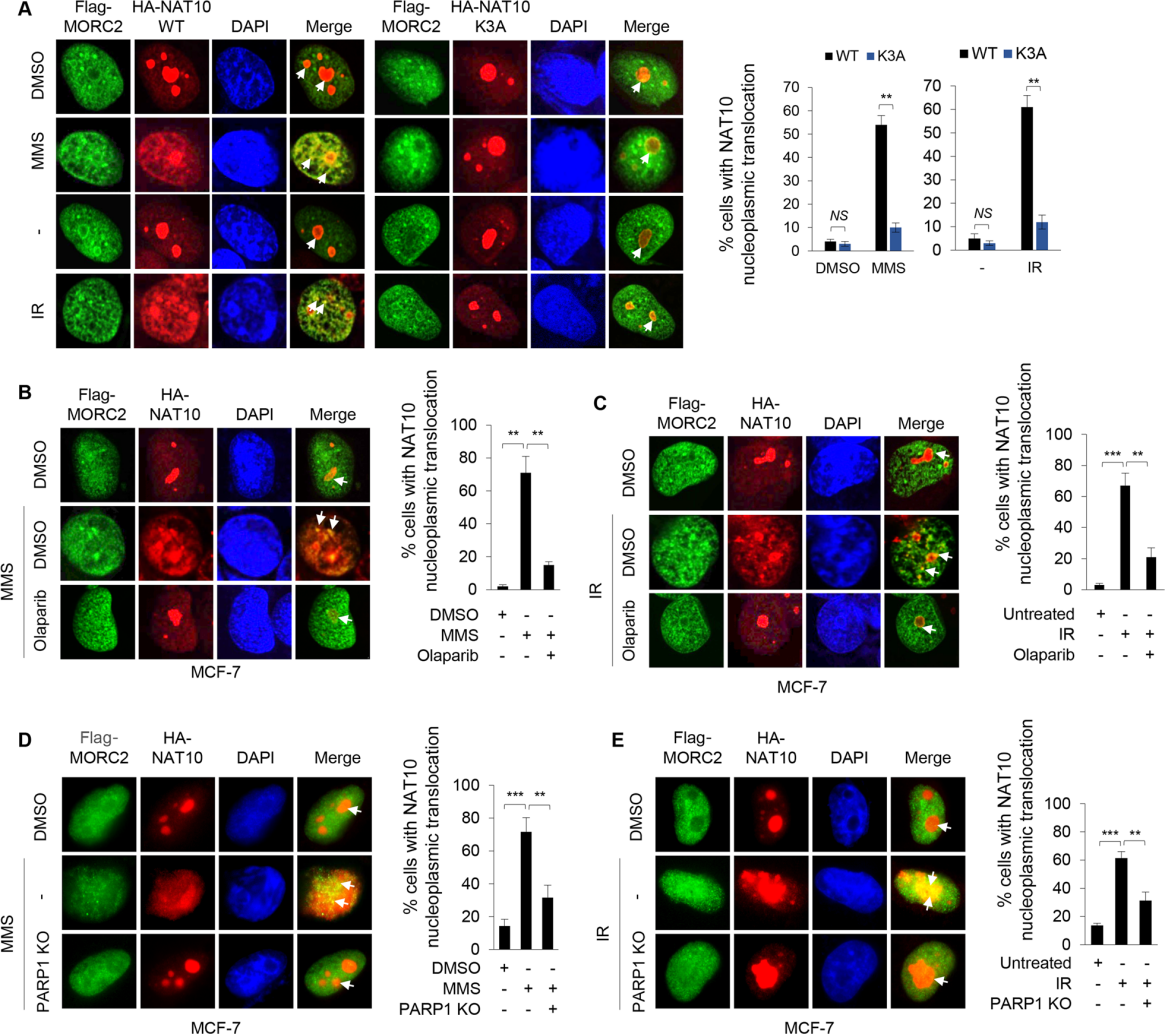
PARylation of NAT10 by PARP1 regulates its nucleoplasmic translocation and co-localization with MORC2. **A** MCF-7 cells were transfected with plasmid DNAs encoding Flag-MORC2, HA-NAT10, or HA-NAT10 K3A. After 48 h of transfection, cells were treated with or without 1 mM MMS for 2 h or 6 Gy IR. IF staining was performed with an anti-Flag (green) or anti-HA (red) antibody. DNA was counterstained with DAPI (blue). Scale bar, 2.5 μm. The quantitative results of cells with NAT10 nucleoplasmic translocation are presented in the right panel. **p < 0.01; *NS*, no significance. **B**, **C** MCF-7 cells were transfected with HA-NAT10 and Flag-MORC2. After 48 h of transfection, cells were pretreated with or without 10 μM Olaparib for 3 h, and then treated with or without 1 mM MMS for another 2 h (**B**) or 6 Gy IR (**C**). IF staining was performed with an anti-Flag (green) or anti-HA (red) antibody. DNA was counterstained with DAPI (blue). Scale bar, 2.5 μm. The quantitative results of cells with NAT10 nucleoplasmic translocation are displayed in the right panel. **p < 0.01; ***p < 0.001. **D**, **E** PARP1-KO MCF-7 cells were transfected with HA-NAT10 and Flag-MORC2. After 48 h of transfection, cells were treated with or without 1 mM MMS for another 2 h (**D**) or 6 Gy IR (**E**). IF staining was performed with an anti-Flag (green) or anti-HA (red) antibody. DNA was counterstained with DAPI (blue). Scale bar, 2.5 μm. The quantitative results of cells with NAT10 nucleoplasmic translocation are displayed in the right panel. **p < 0.01; ***p < 0.001. Arrows indicate the colocalization between MORC2 and NAT10

We recently demonstrated that DNA-damaging agents promote the translocation of NAT10 from the nucleolus to the nucleoplasm and enhance its interaction and co-localization with MORC2 [16]. However, the mechanism by which this occurs remains unexplored. It has been shown that PARP1 is localized in both the nucleoplasm and the nucleolus (approximately 40% of PARP1 in the nucleolus) [44, 45] and is able to regulate DNA damage-induced nucleolar-nucleoplasmic shuttling of genome maintenance factors [46]. As PARP1-mediated PARylation of NAT10 resides within its C-terminal NuLS motif (Fig. 2H), we next determined whether PARP1 is involved in NAT10 nucleoplasmic translocation and the enhanced co-localization between NAT10 and MORC2 following DNA damage. To do this, we transfected MCF-7 cells with Flag-MORC2, HA-NAT10, or HA-NAT10 K3A, and treated them with MMS or IR. Immunofluorescence staining showed that HA-NAT10 enabled to translocate to the nucleoplasm after treatment with MMS and IR, whereas HA-NAT10 K3A still remained mainly in the nucleolus (Fig. 3A), indicating that the PARP1-mediated PARylation is required for NAT10 translocation to the nucleoplasm in response to DNA damage. Moreover, the co-localization between Flag-MORC2 and HA-NAT10 was increased following DNA damage; however, this effect was compromised in cells expressing HA-NAT10 K3A (Fig. 3A).

To confirm these findings, MCF-7 cells were transfected with Flag-MORC2 and HA-NAT10, and then treated with or without MMS and IR in the presence or absence of Olaparib. As shown in Fig. 3B, C, pretreatment of MCF-7 cells with Olaparib impaired MMS- and IR-induced translocation of HA-NAT10 from the nucleolus to the nucleoplasm and, consequently, reduced its co-localization with Flag-MORC2. Consistently, knockout of PARP1 also attenuated MMS- and IR-induced translocation of HA-NAT10 from the nucleolus to the nucleoplasm and reduced its co-localization with Myc-MORC2 (Fig. 3D, E). Collectively, these results suggest that PARP1-mediated PARylation governs the nucleoplasmic translocation of NAT10 and its co-localization with MORC2 in response to DNA damage.

### PARylation of NAT10 by PARP1 controls its interaction with MORC2 in response to DNA damage

**Fig. 4:**
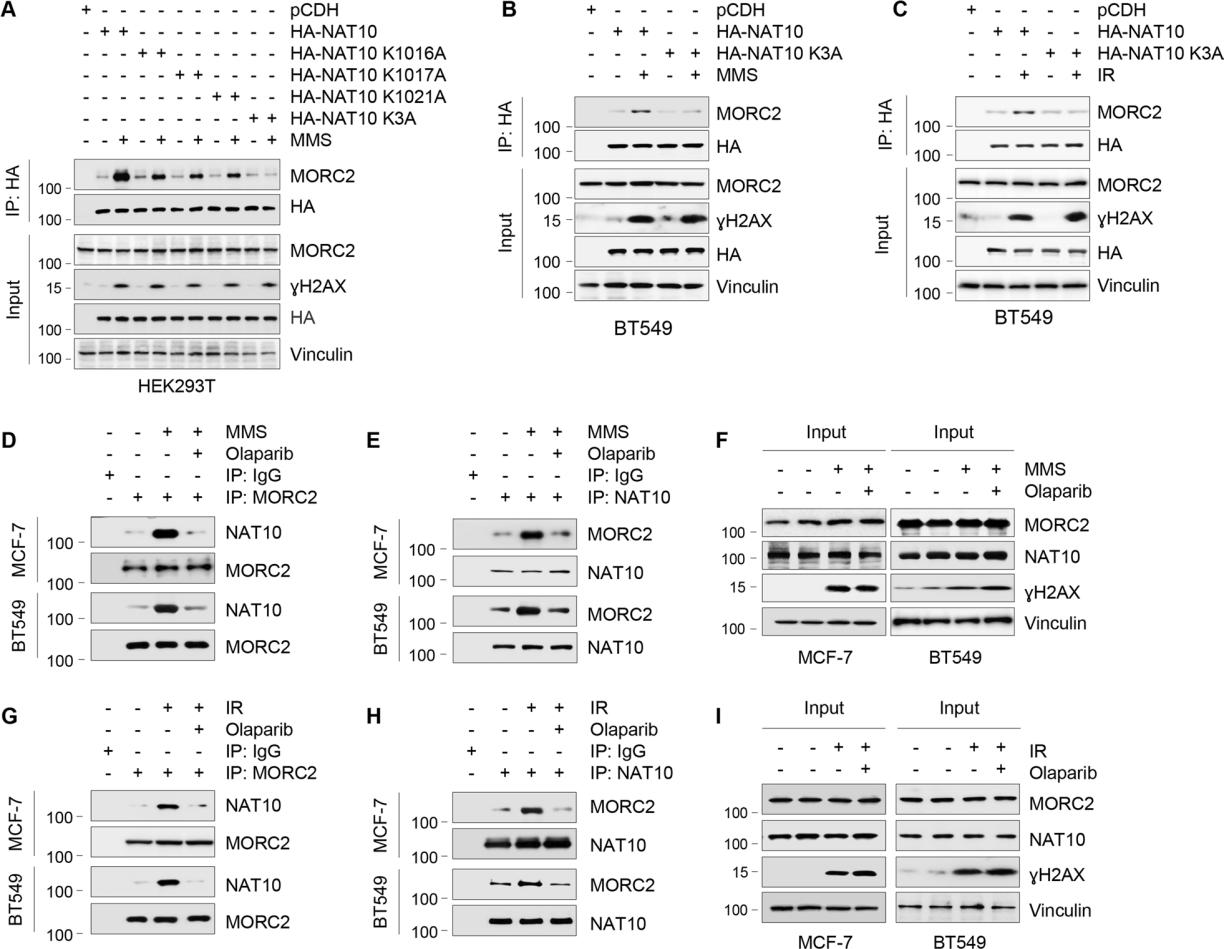
PARylation of NAT10 by PARP1 regulates its interaction with MORC2. **A** HEK293T cells were transfected with the indicated expression vectors. After 48 h of transfection, cells were treated with or without 1 mM MMS for 2 h and subjected to IP and immunoblotting analyses with the indicated antibodies. **B**, **C** BT549 cells were transfected with plasmid DNAs encoding pCDH, HA-NAT10, or HA-NAT10 K3A. After 48 h of transfection, cells were treated with or without 1 mM MMS for 2 h (**B**) or 6 Gy IR (**C**). IP and immunoblotting analyses were performed with the indicated antibodies. **D**–**F** MCF-7 and BT549 cells were pretreated with or without 10 μM Olaparib for 3 h, and then treated with or without 1 mM MMS for another 2 h. The sequential IP and immunoblotting analyses were performed with the indicated antibodies. **G**–**I** MCF-7 and BT549 cells were pretreated with or without 10 μM Olaparib for 3 h, and then treated with or without 6 Gy IR. The sequential IP and immunoblotting analyses were performed with the indicated antibodies

To examine whether PARylation of NAT10 affects its interaction with MORC2, we transfected HEK293T cells with various HA-NAT10 expression vectors (WT, K1016A, K1017A, K1020A, and K3A), and treated them with or without MMS. Sequential IP and immunoblotting analysis revealed that MMS-induced increase in the interaction between NAT10 and MORC2 was significantly impaired in cells expressing HA-NAT10 K3A mutant compared to its WT counterpart and other mutants (K1016A, K1017A, and K1020A) (Fig. 4A). Similar effects were observed in BT549 cells in response to MMS and IR treatment (Fig. 4B, C). Consistent with these results, treatment of MCF-7 and BT549 cells with MMS and IR enhanced the interaction between endogenous MORC2 and endogenous NAT10, which was compromised by the pretreatment of cells with Olaparib (Fig. 4D–I). Collectively, these results suggest that PARP1-mediated PARylation governs the nucleoplasmic translocation of NAT10 and its co-localization and interaction with MORC2 in response to DNA damage. These results indicate that PARylation of NAT10 by PARP1 controls its interaction with MORC2 in response to DNA damage.

### PARylation of NAT10 by PARP1 is required for DNA damage-induced MORC2 acetylation

**Fig. 5:**
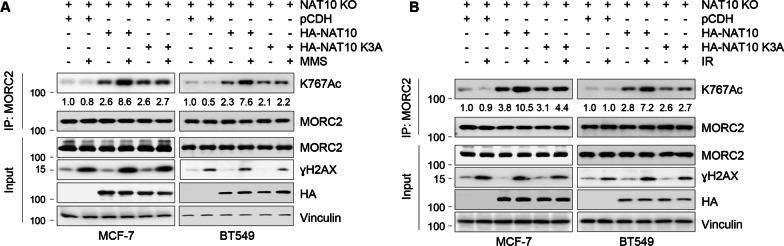
PARylation of NAT10 by PARP1 regulates MORC2 acetylation in response to DNA damage. **A**, **B** NAT10-KO MCF-7 and BT549 cells were transfected with plasmid DNAs encoding pCDH, HA-NAT10, or HA-NAT10 K3A. After 48 h of transfection, cells were treated with or without 1 mM MMS for 2 h (**A**) or 6 Gy IR (**B**), and then subjected to IP and immunoblotting with the indicated antibodies

We recently showed that the interaction between MORC2 and NAT10 is vital for MORC2 acetylation at lysine K767 (K767Ac) in response to DNA damage [16]. To determine whether PARylation of NAT10 affects MORC2 K767Ac, we transfected empty vector pCDH, HA-NAT10, or HA-NAT10 K3A expression vectors into NAT10-knockout (KO) MCF-7 and BT549 cells, and then treated cells with or without MMS or IR. IP assays with an anti-MORC2 antibody, followed by immunoblotting analysis with an anti-K767Ac antibody [16], revealed that cells expressing HA-NAT10 K3A displayed reduced MORC2 K767Ac as compared with HA-NAT10 expressing cells following MMS and IR treatment (Fig. 5A, B).

**Fig. 6:**
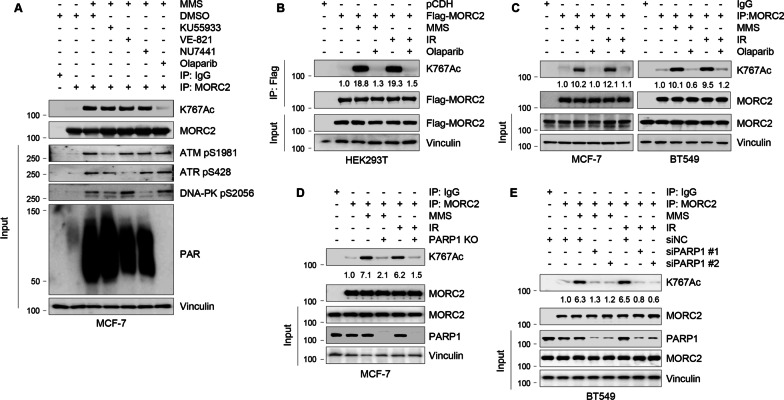
DNA damage induces MORC2 K767Ac in a PARP1-dependent manner. **A** MCF-7 cells were pretreated with or without 10 μM ATM inhibitor (KU-55933), 10 μM ATR inhibitor (VE-821), 10 μM DNA-PKcs inhibitor (NU7441), and 10 μM PARP inhibitor (Olaparib) for 3 h, and then treated with 1 mM MMS for 1 h. IP and immunoblotting analyses were performed with the indicated antibodies. Positive controls for these inhibitors are shown in the input. **B** HEK293T cells stably expressing pCDH and Flag-MORC2 were pretreated with or without 10 μM Olaparib for 3 h, and then treated with 1 mM MMS for another 2 h or 6 Gy IR. IP and immunoblotting analyses were performed with the indicated antibodies. **C** MCF-7 and BT549 cells were pretreated with or without 10 μM Olaparib for 3 h, and then treated with 1 mM MMS for another 2 h or 6 Gy IR. IP and immunoblotting analyses were performed with the indicated antibodies. **D** WT and PARP1-KO MCF-7 cells were treated with or without 1 mM MMS for 2 h or 6 Gy IR. Thereafter, IP and immunoblotting analyses were carried out with the inidicated antibodies. **E** BT549 cells were transfected with siNC or two independent siRNA targeting PARP1 (siPARP1). After 48 h of transfection, cells were treated with or without 1 mM MMS for 2 h or 6 Gy IR. IP and immunoblotting analyses were subsequently performed with the indicated antibodies

ATM, ATR, DNA-PKcs, and PARP1 are the primary sensors of DDR [23, 24]. ATM and DNA-PKcs respond primarily to DNA double-stranded breaks (DSBs), ATR is activated by single-stranded breaks (SSBs) and stalled DNA replication forks [47], and PARP1 is activated by both SSBs and DSBs [24, 25]. We next assessed whether these enzymes have any impact on MORC2 K767Ac after treatment with MMS and IR. As shown in Fig. 6A, pretreatment with the PARP inhibitor (Olaparib), but not the ATM inhibitor (KU55933), ATR inhibitor (VE-821), or DNA-PK inhibitor (NU7441), abolished the MMS-induced upregulation of MORC2 K767Ac, indicating that PARP1 is required for DNA damage-induced MORC2 acetylation. In support of this notion, pharmacological inhibition of PARP1 activity by Olaparib reduced exogenous MORC2 K767Ac in HEK293T cells (Fig. 6B) and endogenous MORC2 K767Ac in MCF-7 and BT549 cells induced by MMS and IR (Fig. 6C). Moreover, depletion of PARP1 in MCF-7 cells using the CRISPR/Cas9 technology decreased MORC2 K767Ac after treatment with MMS and IR (Fig. 6D). This result was further confirmed using two independent siRNAs targeting PARP1 (siPARP1s) in BT549 cells (Fig. 6E). Together, these findings suggest that the DNA damage-induced increase in MORC2 K767Ac is PARP1 dependent.

### PARylation of NAT10 by PARP1 is required for cell survival following DNA damage

**Fig. 7:**
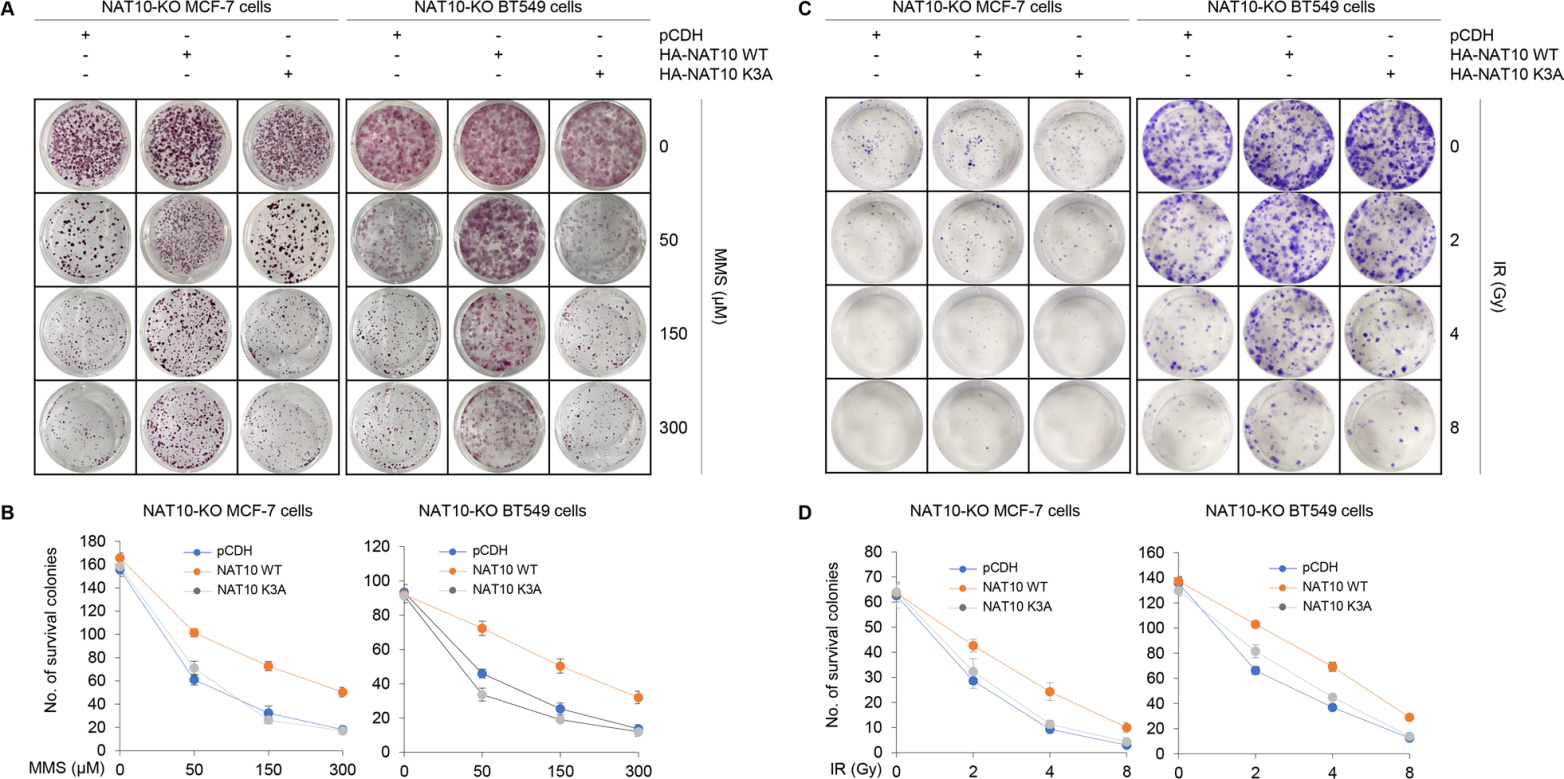
PARylation of NAT10 by PARP1 is required for cell survival in response to DNA damage. **A**, **B** NAT10-KO MCF-7 and BT549 cells stably expressing pCDH, HA-NAT10 WT, or HA-NAT10 K3A were treated with increasing doses of MMS and then subjected to clonogenic survival assays. Representative images of survival colonies are displayed in **A** and the corresponding quantitative results are shown in **B**. **C**, **D** NAT10-KO MCF-7 and BT549 cells stably expressing pCDH, HA-NAT10 WT, or HA-NAT10 K3A were treated with or without 6 Gy IR, and then subjected to clonogenic survival assays. Representative images of survival colonies are displayed in **C** and the corresponding quantitative results are shown in **D**

As NAT10 regulates cellular sensitivity to DNA-damaging agents [16], we proceeded to determine the effects of NAT10 PARylation on the sensitivity of MCF-7 and BT549 cells to MMS and IR. Briefly, we reintroduced the empty vector pCDH, HA-NAT10 WT, and HA-NAT10 K3A into NAT10-KO MCF-7 and BT549 cells, and then carried out clonogenic survival assays in the presence or absence of increasing doses of MMS or after treatment of cells with IR. NAT10-KO MCF-7 and BT549 cells expressing WT NAT10 were found to have decreased cellular sensitivity to MMS compared to NAT10-KO cells expressing empty vector or K3A mutant NAT10 (Fig. 7A, B). Similar results were obtained in these cells treated with IR (Fig. 7C, D). These finding indicates that PARylation of NAT10 by PARP1 is required for its function in response to DNA damage.

## Discussion

**Fig. 8:**
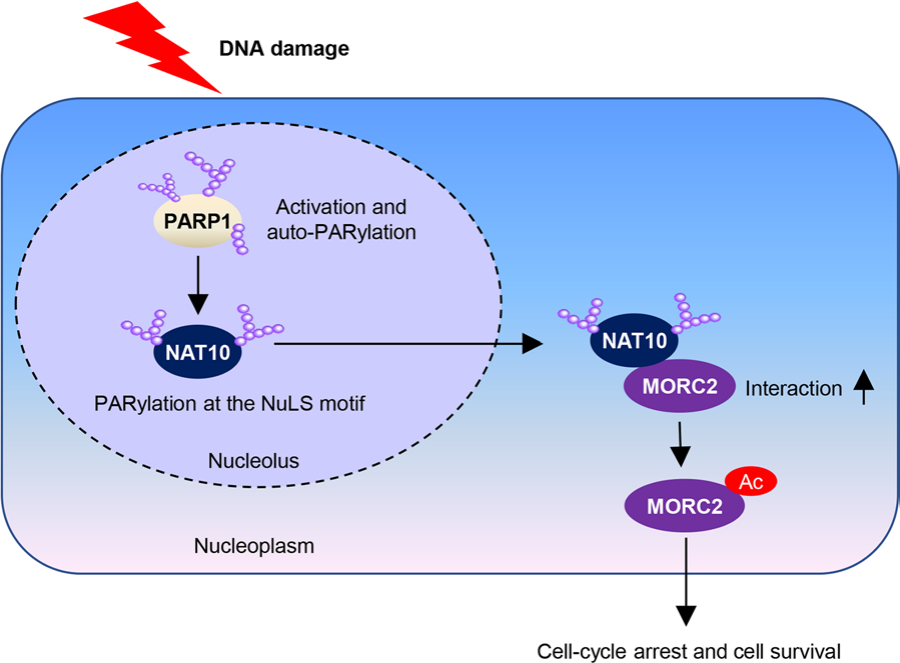
The proposed working model. Activated PARP1 after DNA damage catalyzes the PARylation of NAT10, which is required for the translocation of NAT10 from the nucleolus to the nucleoplasm. NAT10 relocalization increases its co-localization and interaction with its substrate, MORC2, thereby enhancing MORC2 K767Ac in response to DNA damage

In this study, we demonstrated that PARP1 activation after DNA damage catalyzes the PARylation of NAT10 at three conserved lysine residues, which promotes the translocation of NAT10 from the nucleolus to the nucleoplasm. NAT10 translocation increases its co-localization and interaction with its substrate, MORC2, thereby enhancing MORC2 K767Ac and cell survival in response to DNA damage (Fig. 8).

Execution of the DDR relies on a dynamic array of protein modifications, such as phosphorylation, PARylation, and acetylation, which are viewed as important DDR regulators [48, 49]. In the core of DDR, PARP1 triggers early DDR events by PARylating its downstream effector proteins in response to distinct types of DNA damage [24, 25]. Although considerable effort has been made to understand the biological functions of PARylation, only a limited number of PARylation acceptor proteins and few examples of definitive biological roles for site-specific PARylation have been reported to date [36, 50]. In this study, we identified NAT10 as a novel PARylation target of PARP1 (Fig. 1). In support of our findings, a recent high-throughput proteomic study found NAT10 to be PARylated following MMS treatment, but its PARylation sites were not identified [50]. A serial of biochemical analyses further demonstrated that PARP1 catalyzes PARylation of NAT10 at three conserved lysine residues (K1016, K1017, and K1020) within its C-terminal NuLS motif (Fig. 2).

Accumulating evidence shows that some nucleolar proteins undergo DNA damage-specific nucleolar-nucleoplasmic shuttling upon induction of genotoxic stress [46, 51]. A case in point is PARP1, which is localized in both the nucleoplasm and the nucleolus (approximately 40% of PARP1 in the nucleolus) [44]. When cells are exposed to DNA-damaging agents, PARP1 is auto-modified by PARylation and translocates from the nucleolus to the nucleoplasm, where it plays a role in the regulation of DNA repair and cell death induction [44, 45]. PARP1 has also been shown to regulate DNA damage-induced nucleolar-nucleoplasmic shuttling of genome maintenance factors, such as WRN and XRCC1 [46]. Interestingly, other researchers as well as our group recently revealed that NAT10 translocates to the nucleoplasm from the nucleolus when DNA damage is introduced [14, 16]; however, the underlying mechanism has not been determined. We demonstrated for the first time that PARylation of NAT10 by PARP1 is responsible for NAT10 translocation from the nucleolus to the nucleoplasm, thereby resulting in enhanced interaction with MORC2 and thus, MORC2 acetylation (Figs. 3, 4, 5, 6). Consistently, we demonstrated that the DNA-damaging agents, MMS and IR, stimulate MORC2 K767Ac depending on PARP1, but not ATM, ATR, and DNA-PKcs kinases (Fig. 6).

Emerging evidence shows that PARP1 is activated by other mechanisms, in addition to DNA damage [52]. For instance, the interaction between YY1 and PARP1 significantly increases the enzymatic activity of PARP1, thereby regulating downstream gene expression [53]. In addition, PARP1 activity is stimulated by a direct interaction with phosphorylated ERK2 [54, 55]. Therefore, it is necessary to further explore whether these upstream signals enable PARP1 activation, resulting in the translocation of NAT10. In addition, further investigations on other substrates of NAT10, in addition to MORC2, upon activation of PARP1 by a variety of extracellular or intracellular signals should be carried out.

## Conclusions

In summary, the findings of this study suggest that PARylation of NAT10 by PARP1 regulates its translocation and MORC2 acetylation following DNA damage. These findings add another layer of complexity regarding the exact role of PARP1, NAT10, and MORC2 in the cellular response to DNA damage.

## Supplementary Information

**Additional file 1. Table S1.** Chemical inhibitors used in this study. **Table S2.** Information for the expression vectors used in this study. **Table S3.** Primers used for molecular cloning of expression vectors. **Table S4.** siRNA targeting sequences. **Table S5.** Antibodies used in this study.

## Data Availability

All data generated or analyzed during this study are included in this published article.

## Abbreviations

DDR: DNA damage response
IR: Ionizing radiation
MMS: Methyl methanesulfonate
MORC2: MORC family CW-type zinc finger 2
NAT10: N-acetyltransferase 10
PARP1: Poly(ADP-ribose) polymerase 1
PARylation: Poly(ADP-ribosyl)ation
