# Supplementary material for: Poly(ADP-ribosyl)ation of acetyltransferase NAT10 by PARP1 is required for its nucleoplasmic translocation and function in response to DNA damage
Source: Cell Commun Signal. 2022 Aug 19;20:127. doi: 10.1186/s12964-022-00932-1 (PMC9389688; doi:10.1186/s12964-022-00932-1)
Supplement: Supplementary file 2 — Additional file 1. Table S1. Chemical inhibitors used in this study. Table S2. Information for the expression vectors used in this study. Table S3. Primers used for molecular cloning of expression vectors. Table S4. siRNA targeting sequences. Table S5. Antibodies used in this study. [file 12964_2022_932_MOESM2_ESM.docx]

**Additional file 1**

**Supplementary Tables (Table S1-S5)**

**Table S1.** Chemical inhibitors used in this study

**Table S2.** Information for the expression vectors used in this study

**Table S3.** Primers used for molecular cloning of expression vectors

**Table S4.** siRNA targeting sequences

**Table S5.** Antibodies used in this study

**Supplementary Information for**

Liu et al. Poly(ADP-ribosyl)ation of acetyltransferase NAT10 by PARP1 is required for its nucleoplasmic translocation and function in response to DNA damage

**Table S1. Chemical inhibitors used in this study**

| Inhibitors | Vendors | Cat# | Working concentration |
| --- | --- | --- | --- |
| KU55933 | Selleck | S1092 | 10 μM |
| VE-821 | Selleck | S8007 | 10 μM |
| NU7441 | Selleck | S2638 | 10 μM |
| MMS | Sigma | 820775 | 1 mM |
| ADP-HPD | Santa Cruz Biotech | sc-205927 | 5 μM |
| Protease inhibitor cocktail | Bimake | B14002 | 1× |
| Phosphatase inhibitor cocktail | Bimake | B15001 | 1× |

**Table S2. Information for the expression vectors used in this study**

| Plasmids | Sources | Vectors |
| --- | --- | --- |
| Myc-DDK-MORC2 | Origene (RC200518) | pCMV6-Entry |
| Flag-MORC2 | Subcloned | pCDH-CMV-MCS-EF1-Puro |
| GFP-NAT10 | Origene (RG207082) | pCMV6-AC-GFP |
| HA-NAT10 | Subcloned | pCDH-CMV-MCS-EF1-Puro |
| HA-NAT10 K3A | Subcloned | pCDH-CMV-MCS-EF1-Puro |
| GST-NAT10 | Subcloned | pGEX-6P-1 |
| GST-NAT10 1-201 | Subcloned | pGEX-6P-1 |
| GST-NAT10 201-488 | Subcloned | pGEX-6P-1 |
| GST-NAT10 489-753 | Subcloned | pGEX-6P-1 |
| GST-NAT10 754-1025 | Subcloned | pGEX-6P-1 |
| GST-NAT10 754-1025 △754-800 | Subcloned | pGEX-6P-1 |
| GST-NAT10 754-1025 △801-850 | Subcloned | pGEX-6P-1 |
| GST-NAT10 754-1025 △851-900 | Subcloned | pGEX-6P-1 |
| GST-NAT10 754-1025 △901-950 | Subcloned | pGEX-6P-1 |
| GST-NAT10 754-1025 △951-1025 | Subcloned | pGEX-6P-1 |
| GST-NAT10 754-1025 △951-965 | Subcloned | pGEX-6P-1 |
| GST-NAT10 754-1025 △966-980 | Subcloned | pGEX-6P-1 |
| GST-NAT10 754-1025 △981-995 | Subcloned | pGEX-6P-1 |
| GST-NAT10 754-1025 △996-1010 | Subcloned | pGEX-6P-1 |
| GST-NAT10 754-1025 △1011-1025 | Subcloned | pGEX-6P-1 |
| GST-NAT10 754-1025 △1011-1015 | Subcloned | pGEX-6P-1 |
| GST-NAT10 754-1025 △1016-1020 | Subcloned | pGEX-6P-1 |
| GST-NAT10 754-1025-K1016A | Subcloned | pGEX-6P-1 |
| GST-NAT10 754-1025-K1017A | Subcloned | pGEX-6P-1 |
| GST-NAT10 754-1025-D1018A | Subcloned | pGEX-6P-1 |
| GST-NAT10 754-1025-K1020A | Subcloned | pGEX-6P-1 |
| GST-NAT10 754-1025-K3A | Subcloned | pGEX-6P-1 |
| Lenti-Cas9-blast | Addgene (52962) | pFUGW |
| LentiGuide-Puro | Addgene (52963) | lentiGuide-Puro |
| LentiGuide-Puro-NAT10 #1 | Subcloned | lentiGuide-Puro |
| LentiGuide-Puro-NAT10 #2 | Subcloned | lentiGuide-Puro |

**Table S3. Primers used for molecular cloning of expression vectors**

| Plasmids | Primers | Sequences |
| --- | --- | --- |
| Flag-MORC2 | Forward | ACCTCCATAGAAGATTCTAGAGCCACC ATGCTTTGCTTTTTGGATGATGGAG |
|  | Reverse | GATCCATTTAAATTCGAATTCTTACTTA TCGTCGTCATCCTTGTAATC |
| HA-NAT10 | Forward | AAGGGAATTCATGCATCGGAAAAAGGTGGAT |
|  | Reverse | AAGGGGATCCTTAAGCGTAGTCTGGGACGTCGTATGGGTATTTCTTCCGCTTC AGTTTCATA |
| HA-NAT10-K3A | Forward | GAGACAAAGAACGCCGCCGATATGGCCCTGAAGCGGAAG |
|  | Reverse | CTTGTAATCTTTCTTCCGCTTCAGGGCCATATCGGCGGC |
| GST-NAT10 | Forward | AACCGGATCCATGCATCGGAAAAAGGTGGAT |
|  | Reverse | AACCGCGGCCGCCTATTTCTTCCGCTTCAGTTTCAT |
| GST-NAT10-K3A | Forward | GAGACAAAGAACGCCGCCGATATGGCCCTGAAGCGGAAG |
|  | Reverse | GGCCGCCTATTTCTTCCGCTTCAGGGCCATATCGGCGGC |
| GST-NAT10 1-201 | Forward | AACCGGATCCATGCATCGGAAAAAGGTGGAT |
|  | Reverse | AACCGCGGCCGCCTAATCAATGACGAGACACTTCTT |
| GST-NAT10 201-488 | Forward | AACCGGATCCGACCAGCTCAACATCCTGC |
|  | Reverse | AACCGCGGCCGCCTAATCCAGGCACAGCAAGTCAT |
| GST-NAT10 489-753 | Forward | AACCGGATCCTGCCTCAACATCACTCGGAT |
|  | Reverse | AACCGCGGCCGCCTAATCATGCTGAAGACGCTCAC |
| GST-NAT10 754-1025 | Forward | AACCGGATCCGATGAGGATGAGGCTGACC |
|  | Reverse | AACCGCGGCCGCCTATTTCTTCCGCTTCAGTTTCAT |
| GST-NAT10 754-1025 △754-800 | Forward | CGGGATCCGATGAGGATGAGGCTGAC |
|  | Reverse | TAAAGCGGCCGCCATGTTCCTGTTCTGAAT |
| GST-NAT10 754-1025 △801-850 | Forward | CGGGATCCGGGAAGCCAGCCCAGCCT |
|  | Reverse | TAAAGCGGCCGCGAAATAGATGCGAGAGAT |
| GST-NAT10 754-1025 △851-900 | Forward | CGGGATCCCTGAACCAGCTGGGGGAC |
|  | Reverse | TAAAGCGGCCGCGCGGATGATCCGGTTGAA |
| GST-NAT10 754-1025 △901-950 | Forward | CGGGATCCAAAGTTGTGAAGCTATTT |
|  | Reverse | TAAAGCGGCCGCCTTCTTGTGTTTCTCCTG |
| GST-NAT10 754-1025 △951-1025 | Forward | CGGGATCCGAAGTAGGGAAGCTGAAG |
|  | Reverse | TAAAGCGGCCGCTTTCTTCCGCTTCAGTTT |
| GST-NAT10 754-1025 △951-965 | Forward | GAATTTCAGGAGAAACACAAGAAGCGTGGGGACGAT |
|  | Reverse | ATTCCACTCTTCATCGTCCCCACGCTTCTTGTGTTT |
| GST-NAT10 754-1025 △966-980 | Forward | ATGGACCTCTCTGAATACATAATCCCGAACGCCTCG |
|  | Reverse | CAGGCTGATGATCGAGGCGTTCGGGATTATGTATTC |
| GST-NAT10 754-1025 △981-995 | Forward | AATGAAGTTTTGAACAAAGCTGGGTTAGAGGCCAAA |
|  | Reverse | TTTGGGTTCTTGTTTGGCCTCTAACCCAGCTTTGTT |
| GST-NAT10 754-1025 △996-1010 | Forward | CTGAAAAGTGACAAGAAAAGGAAGAGAGAGACAAAG |
|  | Reverse | ATCTTTTTTGTTCTTTGTCTCTCTCTTCCTTTTCTT |
| GST-NAT10 754-1025 △1011-1025 | Forward | AAACAGAGCAAGAAGTTGAAGAACTAGGCGGCCGCA |
|  | Reverse | AGTCAGTCACGATGCGGCCGCCTAGTTCTTCAACTT |
| GST-NAT10 754-1025 △1011-1015 | Forward | AAACAGAGCAAGAAGTTGAAGAACAAAAAAGATATG |
|  | Reverse | CCGCTTCAGTTTCATATCTTTTTTGTTCTTCAACTT |
| GST-NAT10 754-1025 △1016-1020 | Forward | TTGAAGAACAGAGAGACAAAGAACCTGAAGCGGAAG |
|  | Reverse | GGCCGCCTATTTCTTCCGCTTCAGGTTCTTTGTCTC |
| GST-NAT10 754-1025 △1021-1025 | Forward | ACAAAGAACAAAAAAGATATGAAATAGGCGGCCGCA |
|  | Reverse | AGTCAGTCACGATGCGGCCGCCTATTTCATATCTTT |
| GST-NAT10 754-1025 K1016A | Forward | AAGAACAGAGAGACAAAGAACGCCGCCGATATGAAACTG |
|  | Reverse | TTTCTTCCGCTTCAGTTTCATATCGGCGGCGTTCTTTGT |
| GST-NAT10 754-1025 K1017A | Forward | AAGAACAGAGAGACAAAGAACAAAGCCGATATGAAACTG |
|  | Reverse | TTTCTTCCGCTTCAGTTTCATATCGGCTTTGTTCTTTGT |
| GST-NAT10 754-1025 D1018A | Forward | AACAGAGAGACAAAGAACAAAAAAGCCATGAAACTGAAG |
|  | Reverse | CTATTTCTTCCGCTTCAGTTTCATGGCTTTTTTGTTCTT |
| GST-NAT10 754-1025 K1020A | Forward | GAGACAAAGAACAAAAAAGATATGGCCCTGAAGCGGAAG |
|  | Reverse | GGCCGCCTATTTCTTCCGCTTCAGGGCCATATCTTTTTT |

**Table S4. siRNA targeting sequences**

|  | Primers | Sequences |
| --- | --- | --- |
| siPARP1 #1 | Forward | GAGACCCAAUAGGCUUAAUTT |
|  | Reverse | AUUAAGCCUAUUGGGUCUCTT |
| siPARP1 #2 | Forward | GAGGAAGGUAUCAACAAAUTT |
|  | Reverse | AUUUGUUGAUACCUUCCUCTT |

**Table S5. Antibodies used in this study**

| Antibodies | Vendors | Cat# | Hosts | Working concentration | |
| --- | --- | --- | --- | --- | --- |
| NAT10 | Abcam | ab194297 | R | | 1: 1500 (WB)  1:150 (IHC) |
| Phospho-DNA-PKcs (S2056) | Abcam | ab124918 | R | | 1: 1500 (WB) |
| MORC2 | Bethyl | A300-149 | R | | 1: 1000 (WB) |
| HA | CST | S3724 | R | | 1: 1500 (WB)  1: 500(IF) |
| phospho-ATM (S1981) | CST | S5883 | R | | 1: 1500 (WB) |
| phospho-ATR (S428) | CST | S2853 | R | | 1: 1500 (WB) |
| γH2AX | CST | S7631 | R | | 1: 1500 (WB) |
| Mouse IgG,Isotype Control | CST | 5415 | M | | 1: 1000 (WB) |
| Rabbit IgG,Isotype Control | CST | 3900 | R | | 1: 1000 (WB) |
| Mouse IgG,HRP-linked | CST | 7076 | M | | 1: 1000 (WB) |
| Rabbit IgG,HRP-linked | CST | 7074 | R | | 1: 1000 (WB) |
| Mouse IgG  (Alexa Fluor 555 Conjugate) | CST | 4409 | M | | 1: 500 (IF) |
| Rabbit IgG  (Alexa Fluor 488 Conjugate) | CST | 4412 | R | | 1: 500 (IF) |
| GST | GNI | GNI4110-GT | M | | 1: 1000 (WB) |
| PARP1 | Santa Cruz | sc-8007 | M | | 1: 1000 (WB) |
| Flag | Sigma | F3165 | M | | 1: 1500 (WB)  1: 500 (IF) |
| Vinculin | Sigma | V9131 | M | | 1: 3000 (WB) |
| PAR | Trevigen | 4335-MC-100 | M | | 1: 1000 (WB) |
| MORC2 K767Ac | Customer made | - | R | | 1: 1000 (WB) |

*Notes: M, mouse; R, rabbit.*
